# Oral health-related quality of life outcomes in a randomized clinical trial to assess a community-based oral hygiene intervention among adults living in low-income senior housing

**DOI:** 10.1186/s12955-021-01859-w

**Published:** 2021-09-28

**Authors:** Susan Reisine, Jean J. Schensul, Apoorva Salvi, James Grady, Toan Ha, Jianghong Li

**Affiliations:** 1 Division of Behavioral Sciences and Community Health, School of Dental Medicine, University of Connecticut, 263 Farmington Avenue, Farmington, CT 06030 USA; 2 Institute for Community Research, Two Hartford Square West, Suite 100, 146 Wyllys St., Hartford, CT 06106 USA; 3 Oregon Health and Science University, 3181 S.W. Sam Jackson Park Road, Portland, OR 97239 USA; 4 Department of Community Medicine, University of Connecticut School of Medicine, 195 Farmington Avenue, Farmington, CT 06030 USA; 5 Department of Infectious Diseases and Microbiology, Graduate School of Public Health, University of Pittsburgh, 130 De Soto St., Pittsburgh, PA 15261 USA

**Keywords:** Oral health related quality of life, General Oral Health Assessment Index, Oral hygiene, Clinical trial

## Abstract

**Background:**

Quality of life outcomes have been used frequently in clinical trials of oral health interventions. This study assessed the effects of a randomized trial on oral health related quality of life comparing an individual-based oral hygiene intervention to a community-based intervention.

**Methods:**

Participants were recruited from six low-income senior housing residences. Buildings were randomly assigned to receive the individual-based intervention followed by the community-based intervention or to receive the community-based intervention followed by the individual intervention. Participants’ oral hygiene was assessed at baseline (T0), one month after the first intervention (T1) and one month after the second intervention (T2) and six months after the T2 assessment (T3). Oral hygiene was measured by the Gingival Index (GI) and Plaque scores (PS). Surveys collected data on beliefs, attitudes, behaviors and self-reported health status at T0, T1 and T2. Only oral hygiene and quality of life, measured by the General Oral Health Assessment Index (GOHAI), was assessed at all time points. general linear mixed models (GLMM) were used to assess changes in GOHAI over time, the interaction of condition by time and the contribution of psychosocial, behavioral, health status and background variables to changes in GOHAI.

**Results:**

331 people completed T0 assessments; 306 completed T1; 285 completed T2 and 268 completed T3. Scores on GOHAI at T0 ranged from 10 to 48 with a mean of 39.7 (sd = 7.8) and a median of 42. At T1, mean GOHAI was 40.7 (sd = 8.2), at T2 mean GOHAI was 41.1 (sd = 7.8) and at T3, GOHAI was 42.3 (sd = 8.2). GLMM showed that GOHAI improved significantly from T0 to T3 (*p* = 0.01) but the time by intervention interaction was not significant indicating that both interventions were effective in improving GOHAI but one intervention was not better than the other. Ethnicity, health status, worries, self-efficacy, number of missing teeth and symptoms of dry mouth were related to improvements in GOHAI. Neither GI nor PS were related to GOHAI.

**Conclusions:**

The participants reported relatively good oral health related quality of life which improved significantly over time. Improvement occurred among all participants regardless of condition, suggesting that either intervention would be effective in future studies.

*Trial Registry*: Clinicaltrials.gov, Clinical Trials ID #NCT02419144; Title: A Bi-level Intervention to Improve Older Adult Oral Health Status; Registered 04/07/2015 URL: https://register.clinicaltrials.gov/prs/app/action/SelectProtocol?sid=S0005H9X&selectaction=Edit&uid=U0000KBK&ts=2&cx=-rajj5q

## Introduction

In line with the WHO definition of health as “a complete state of physical, mental, and social well-being and not just the absence of disease” [1], quality of life recently has been incorporated into the professional conceptual framework of oral health [2], lending support to the notion that clinical indicators alone are not sufficient to assess oral health in populations or outcomes in clinical trials. The literature on Oral Health-Related Quality of Life (OHRQOL) has exploded since the 1990s [3] as interest in patient-centered outcomes has expanded. Most studies in this area have focused on epidemiological studies of population OHRQOL, such as the NHANES [4], Medicare Primary and Consumer-Directed Care (PCDC) Demonstration [5], the UK Adult Dental Health Survey [6], National Dental Telephone Interview Survey [7], and The Health Risk Appraisal for Older Persons study in England [8]. Although there are many measures of OHRQOL [9], these studies either used the Oral Health Impact Profile (OHIP) [10] or the General Oral Health Assessment Index (GOHAI) [11] to assess OHRQOL. Levels of OHRQOL vary from relatively good OHRQOL in these population surveys [6] to moderate levels [8] to low quality of life [7] to poor OHRQOL, among clinical populations [12–14]. These studies have consistently shown that older age, higher education, non-minority status and higher socioeconomic status are associated with better OHRQOL.

Clinical indicators of oral health status, including periodontal disease, dental caries [6, 15], missing teeth and xerostomia, and their association with OHRQOL have been investigated, as well. Although some studies show an association between periodontal disease, gingivitis and its treatment [14, 16] and OHRQOL, other studies find no association between periodontal disease and OHRQOL [6, 17]. The number of missing teeth seems to be more important in influencing OHRQOL; several studies demonstrate a strong and consistent association between number of missing teeth and OHRQOL—more missing teeth are associated with poorer OHRQOL [4, 5, 7, 18]. Dental caries also has a significant effect on OHRQOL in that a higher number of carious teeth [6, 15] and higher DMFT [15] result in worse OHRQOL. Studies of older adults often find that xerostomia or dry mouth is a common oral health problem largely because of the effects of medications on salivation [19]. Some studies estimate that one in five older adults experience dry mouth [19] with corresponding ill effects on quality of life [20].

OHRQOL is well accepted as an important construct in defining oral health, and has been used as an outcome measure in dental clinical trials among adults. Studies using OHRQOL as an outcome range from prosthodontics [21], endodontics [22] periodontology [23], health care delivery interventions [24], oral hygiene [25] and psychosocial [12] and educational interventions [14] and xerostomia [25].

## Theoretical model

A limitation of much of the literature on clinical trials in oral health interventions is that most trials do not have a theoretical model to structure the intervention [26]. The theoretical model that informs this study is based on an adaptation of Fishbein’s Integrative Model of Behavior Change [27] and Bandura’s concept of self-efficacy [28]. The model includes sociodemographic factors, general health, mental health and oral health factors; the interventions; cognitive and behavioral factors which offer possible explanations for behavior change. These previous factors lead to intentionality, which is the critical factor in the model. See Schensul et al. [29] for more detailed discussion of the model.

Another unique aspect of this trial is that this is a bi-level intervention. One intervention is aimed at the individual, tailored to  identified areas of deficiencies in the theoretical model which we term Adapted Motivation Interviewing. The other intervention is a norms-based intervention at the community level, aimed at changing beliefs, attitudes and behaviors through building-based oral hygiene campaigns.

The purpose of this paper is to evaluate (1) whether OHRQOL improves as an outcome in a clinical trial of an individual-based Adapted Motivational Interviewing intervention (AMI) compared to a community-based campaign intervention among adults living in low-income senior housing; (2) whether receiving the AMI intervention first followed by the campaign is more effective in improving OHRQOL than receiving the campaign first followed by the AMI intervention; and (3) whether clinical, demographic and psychosocial/behavioral variables affect changes in OHRQOL.

### Hypotheses

1. Individuals will have significant improvements in OHRQOL in both interventions in short term (T1), medium term (T2) and long-term (T3) outcomes.
2. The Individual-based intervention will produce better short term (T1), medium term (T2) and long-term (T3) improvements in OHRLQOL than those in the community-based Campaign intervention.

## Methods

This study was a randomized cluster design. We recruited six low-income senior housing buildings to the study which then were randomly assigned by the biostatistician to either receive the individual-based intervention, Adapted Motivational Interviewing (AMI), followed by the community-based campaign intervention (Condition 1), or to first receive the campaign intervention followed by the AMI (Condition 2) (see below for descriptions of the interventions). The protocol and methods have been published in Schensul et al. [29] and on our website, projectgoh.com. The website offers detailed information on the interventions, procedures and design. A cross-over design was used to assess whether AMI was more effective than the campaign intervention overall and whether sequencing of the interventions mattered. Participants were assessed at baseline, T0; a month after each first intervention, T1; a month after the second intervention, T2; and at T3, six months after the T2 assessment,

### Eligibility criteria

Residents of the building who met the following inclusion and exclusion criteria were recruited to the study. *Inclusion criteria*: (a) Male or female; (b) being 18 years and above. At the request of the funding agency, we limited the number of people less than 62 years of age to eight participants in the last two buildings; (c) being a permanent resident of the building; (d) without a conservator; (e) judged competent to participate; (f) at least two natural teeth present in the dentition. *Exclusion criteria*: (a) cognitively incompetent to give informed consent; (d) history of infective endocarditis, prosthetic cardiac valve replacement in the past 6 months, insertion of an arterial stent in past 6 weeks, myocardial infarction in past 6 weeks; (e) joint replacement surgery, or currently on dialysis.

### Study location and data collection

The study took place in each of the six buildings. Once residents agreed to be in the study, they completed an informed consent process and received an oral exam to assess gingival inflammation and plaque levels. Another visit was scheduled to complete a survey that collected data on demographics, beliefs, attitudes and oral health behaviors using the using the computer-based Questionnaire Development System (QDS) [30]. Surveys were administered in either English or Spanish based on the preference of the participant. These two visits constituted T0. The clinical assessment and the survey were repeated at T1 and T2, and the clinical assessment and GOHAI were completed at T3.

### The interventions

The interventions consisted of an individual-based counselling session, Adapted Motivational Interviewing (AMI) and a building-based campaign consisting of three health fairs. Both interventions were administered in English and Spanish.

### AMI—individual-based intervention

The AMI intervention was a tailored counseling session. Tailoring was based on responses to the cognitive/behavioral variables in the theoretical domains in Fishbein’s MI and Bandura’s Integrated Model of Behavior Change described above. Cut-offs, established during the pilot study [31] identified areas of need for intervention. If the participant scored below the cut-off, that variable was addressed during the AMI counseling session. Sessions began with a general exploration of whether participants had questions about the study and their concerns about their oral health. Following this introduction, the interventionist addressed the areas from the survey that needed attention and addressed any other concerns. This was followed by oral hygiene instruction though videos of correct brushing and flossing techniques. Participants were shown their charts from the clinical exam which illustrated where plaque existed. The interventionist demonstrated brushing and flossing skills on a typodont and participants then demonstrated skills on a typodont, were scored and given feedback. The participant and counselor developed an action plan which both signed. One hundred and sixty five in Condition 1 completed the AMI at T1 and 140 in Condition 2 completed the AMI at T2.

### Building-based campaigns

The campaigns were organized by residents who volunteered to plan and carry out the campaign by joining the Campaign Committee with support of the research staff. Committees consisted of 6–10 people in each building. Members of the Campaign Committee participated in a 12 week training program on research methods offered by the intervention staff on theoretical cognitive/behavioral domains and research ethics, persuasive messaging related to each of the domains, and campaign organization. The Campaigns consisted of three health fairs conducted about one month apart in common areas in each building. The health fairs lasted about two hours and residents rotated through 12 oral hygiene motivational stations representing the cognitive/behavioral domains of the IM. The stations featured games, hands-on oral hygiene instruction and oral health information. Oral health professionals made presentations followed by a question and answer period. The health fairs were open to all building residents. See Schensul et al. [29] and our website, projectgoh.com, for additional details on the interventions. Seventy-two people in Condition 1 and 76 people in Condition 2 attended at least one fair.

### Primary outcome measure—oral health related quality of life (OHRQOL)

OHRQOL was measured by the General Oral Health Assessment Index (GOHAI), a commonly used 12-item measure initially developed for older adults that has been used with low income and minority populations [11]. Participants rated the frequency of negative (nine items) and positive impacts (three items) of their teeth, dentures and gums in the previous three months from 0, always, to 4, never. Three positive impact items were reverse-coded. Total scores ranged from 0, poor OHRQOL, to 48, best OHRQOL. Cronbach’s alpha = 0.80.

### Sample size

Sample size was determined based on data from the pilot study [31]. The mean difference in GI in the pilot study was 0.66 (sd = 0.60) and we estimated the effect size for GI at 1.1 For PS, there was a mean difference of 0.24 (sd = 0.35) thus estimating the effect size at 0.74. Sample size was computed using a two-group t-test and two sided alpha = 0.05. For each measure, we determined that a sample of 123 in each group would have 99% power. Assuming a 75% retention rate, a total of 360 participants with a study/cluster size of 60 in each building, would have adequate power to detect differences in the interventions.

## Measures

### Clinical measures of oral hygiene and oral health

#### Oral hygiene

Oral hygiene was assessed by gingival inflammation using the Gingival Index (GI) [32] and by presence of plaque measured by Plaque Scores (PS) [33]. GI measures inflammation of the gums on six sides of each tooth present in the mouth. Inflammation is scored on a four-point scale: 0, no inflammation to 3 overt inflammation. The score on each gum surface surrounding each tooth is summed and then divided by the total number of teeth present for an average score for each participant. PS measured the presence or absence of plaque on six tooth surfaces after application of erythrosine disclosing solution. PS is the percent of teeth with plaque present.

Two hygienists conducted the clinical assessments after extensive training and calibration. Calibration was completed T0, T1, T2 and T3. At T0, as training continued, the best Kappa score was 0.78 for PS and 0.54 for GI. Kappa improved to a range of 0.77 to 0.94 for PS and 0.72 to 1.00 for GI at T1. For T2, Kappa for GI ranged from 0.69 to 0.79 and for PS Kappa ranged from 0.60 to 0.79. For T3, Kappa ranged from 0.57 to 0.78 for PS and 0.57 to 0.78 for GI. Assessors were not blinded to condition.

#### Missing teeth

The dental hygienists recorded the number of missing teeth during the clinical exam. The mean number of missing teeth in the sample at baseline was 14.1 (sd = 7.0).

#### Xerostomia

Xerostomia is the subjective feeling of dry mouth and was assessed by self-report in the survey. The measure developed by Fox and colleagues [34] was used to assess dry mouth and consists of eight items with yes/no response. A scale was calculated by summing the total number of items to which the participants responded yes.

All measures were administered in English and Spanish using standard translation/backtranslation methods required by the UCONN IRB.

#### Demographic and background characteristics

Covariates also included background factors such as demographic characteristics, general health status, oral health status and mental health. Demographic factors included age, gender, marital status, income, education and health insurance. General health was measured in two ways. Respondents were asked whether they had been diagnosed with any of 13 conditions listed in the survey and whether these conditions interfered with daily activities. One variable consisted of the number of diagnosed conditions and the other variable was the count of the number of conditions that affected daily activities [35]. Participants rated oral health status on a four-point scale [36], excellent, good, fair and poor. This variable was dichotomized for the analysis as excellent/good and fair/poor. The Center for Epidemiologic Studies Depression Scale -Short Form (CESD-SF), a measure of the frequency of depressive symptoms [37], assessed mental health status.

### Cognitive/behavioral factors

The cognitive behavior variables are based on Fishbein’s IM and Bandura’s theory of self-efficacy (see Schensul et al. [29] for detailed discussion of the model and cognitive behavioral variables). These variables have the potential to provide insights into the mechanisms accounting for behavior change. Six cognitive variables and three behaviors were measured at T0, T1 and T2 by the survey. The Alpha scores are from T0 data.

### Cognitive variables

#### Oral health self-efficacy [38]

This variable assessed the participant’s belief in his/her ability to care for teeth. It is a five-item Likert scale rating agreement with the five statements on a scale of 1–4; higher scores indicated higher self-efficacy or participants’ belief in their ability to care for teeth. Cronbach’s alpha was 0.63.

#### Fears of oral diseases

This scale evaluated the level of fear participants had about developing oral health problems. The scale consisted of four items and participants rated how afraid they were of developing four oral health problems on a four-point scale from very (1) to not at all (4). Higher scores indicated less fear. Cronbach’s alpha was 0.82.

#### Intentionality [41]

Intentionality is a key component of the IM consisting of ten items rating intention to perform preventive oral health behaviors. Intention was rated as 0, no possibility to 2, good possibility on ten behaviors. Higher scores indicate greater intentionality. Cronbach’s alpha was 0.72.

#### Locus of control [38]

Locus of control measures the belief in the ability to control his or her own oral health. A seven-item Likert scale assessed locus of control by rating agreement with the seven statements on a scale of 1–4, strongly agree (1) to strongly disagree (4). Higher scores indicated greater locus of control. Alpha was 0.72.

#### Importance of oral health behavior—oral health norms [39]

Oral health norms assesses the belief in the importance of normative preventive oral health behaviors. Participants rated the importance of nine preventive oral health behaviors on a scale of 1, not important at all to 4, very important. Cronbach’s alphas was 0.67.

#### Oral hygiene self-management worries scale (OHWSMS) [40]

This validated scale, created for this study, evaluates participants’ worries about taking care of their teeth. It consists of 19 items related to oral hygiene behaviors, rated on how worried participants were about each item. The responses were 1, very worried to 4, not worried at all. Higher scores indicate less worry. Cronbach’s alpha was0.93.

Behavioral variables included sugar intake, brushing and flossing frequency.

#### Sugar intake

Participants rated the frequency of consuming five foods high in sugar and starch from 0, never, to 4, more than five times a day. Participants reported Brushing frequency and Flossing frequency. These behaviors were assessed as once or more per day or less than once a day.

### Statistical analysis

The initial analyses were descriptive with frequencies, means and standard deviations of the variables summarized to characterize the sample. Kruskal-Wallace tests and Spearman correlation coefficients were used to assess the bivariate relationships between GOHAI and demographic characteristics and cognitive behavioral variables at baseline because GOHAI was skewed to positive scores. Repeated measures general linear mixed models (GLMMs) were used to assess the relationship of GOHAI with intervention, time, the (intervention × time) interaction plus the explanatory variables for demographics and cognitive/behaviors variables. These models were fit in SAS® [41] using the MIXED procedure. A model with GOHAI as a binary outcome (median as cut point) using general estimating equations (GEE) as a sensitivity analysis yielded the same set of predictors with similar interpretations.

## Results

**Fig. 1:**
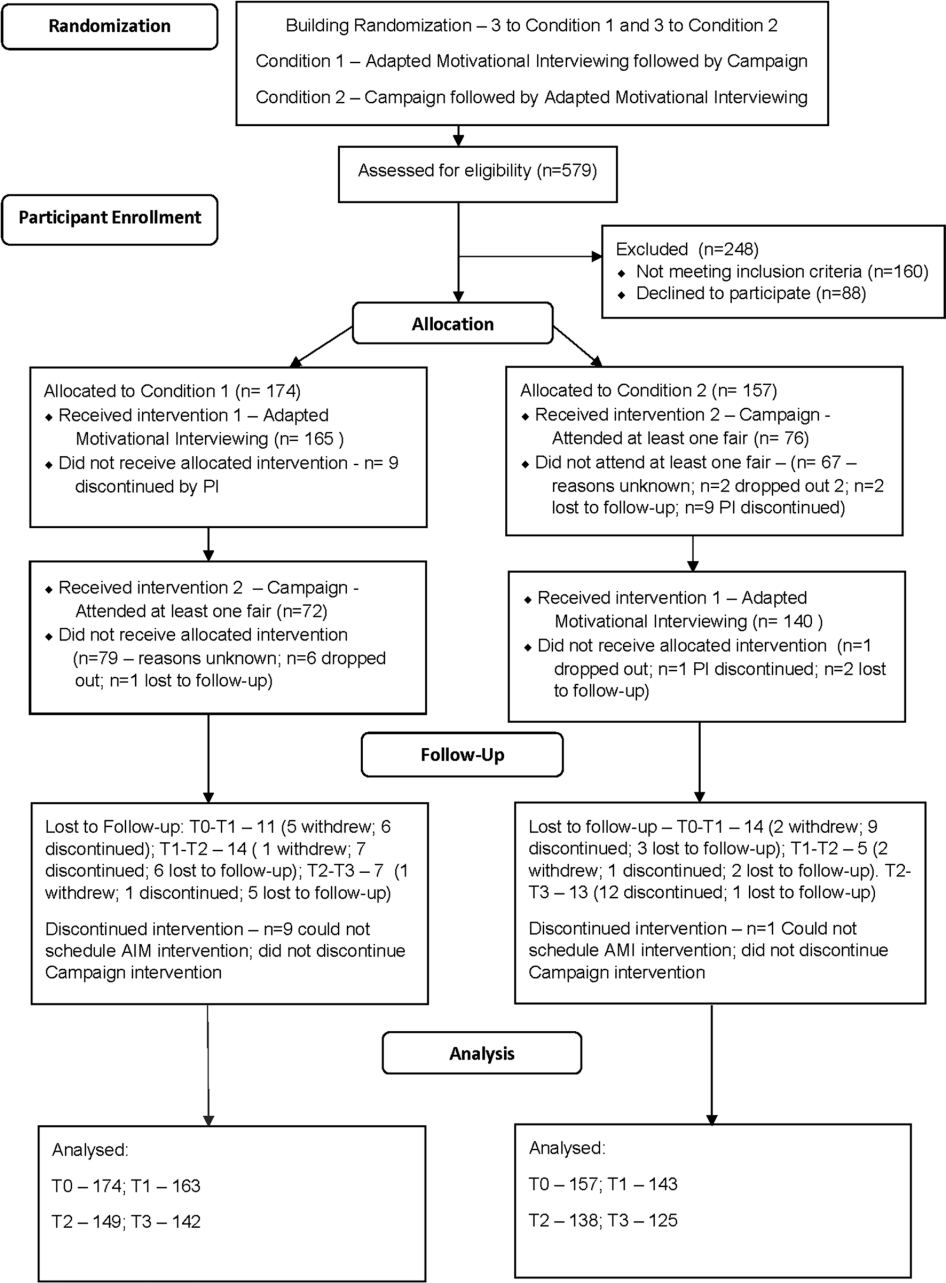
Workflow diagram

The flowchart in Fig. 1 illustrates the research process. Four hundred and nineteen people met the eligibility criteria. Eighty-eight individuals were either lost to follow-up prior to enrollment or declined to participate. Three hundred and thirty-one people completed T0; 306 completed T1 assessments (92.4% retention rate), 285 completed T2 assessments (86.1% retention rate) and 268 completed T3 assessments (81%). Scores on GOHAI at T0 ranged from 10 to 48 with a mean of 39.7 (sd = 7.8) and a median of 42. The distribution of scores at each time point was skewed to higher, more positive scores. At T1, mean GOHAI was 40.7 (sd = 8.2), at T2 mean GOHAI was 41.1 (sd = 7.8) and at T3, GOHAI was 42.3 (sd = 8.2).

Table 1 presents the descriptive characteristics of the sample and self-reported oral hygiene behaviors at T0. Table 1 also presents data on the relationship between the demographic, health status and oral hygiene variables and the mean (sd) scores on GOHAI. Kruskal-Wallace test assessed group differences. None of the demographic covariates, health insurance or oral hygiene behaviors were significantly associated with GOHAI—all *p* values were greater than 0.10. However, three health status variables were significantly related to GOHAI—those with more diagnoses that interfered with daily activities, those who had higher CESD-SF scores and those who reported worse oral health status had lower GOHAI scores.

**Table 1:** Descriptive, health and oral hygiene characteristics of the sample at baseline (T0) and their relationship to GOHAI (n = 331)

	Percent of total sample	Mean (SD) GOHAI Summary Score Sample Mean = 39.7 (sd = 7.8)	*p* value Kruskal–Wallis H test
*Background characteristics*			
Gender			0.84
Male	42.0	39.7 (7.9)	
Female	58.0	39.6 (7.7)	
Age			0.20
< 62	31.1	37.9 (8.5)	
> = 62	68.9	40.4 (7.3)	
Race/ethnicity			0.51
Hispanic	58.3	38.9 (8.3)	
Black Not Hispanic	23.0	40.1 (7.8)	
White Not Hispanic and others	18.7	39.1 (7.0)	
Education			0.61
Less than high school	47.7	39.9 (8.2)	
Completed high school or more	52.3	39.5 (7.4)	
Marital status			0.12
Single	30.2	38.9 (8.3)	
Married/living as married	16.1	38.4 (8.8)	
Separated/divorced	35.0	40.1 (6.5)	
Widowed	18.7	41.0 (8.1)	
Income			0.99
< $900	51.6	39.4 (8.1)	
> = $900	48.4	39.9 (7.6)	
*Health status variables*			
Health insurance			0.49
Medicaid & Medicare + Medicaid	51.0	39.1 (8.0)	
Medicare only	13.0	39.5 (7.6)	
Other	36.0	42.2 (6.1)	
Number of diagnoses (Mean = 3.8 (sd = 1.4); median = 4)			0.27
0–4	67.4	40.5 (7.4)	
4 +	32.6	37.9 (8.2)	
Number of diagnoses that interfere with daily activities			0.01
0	42.0	42.4 (6.3)	
1 +	58.0	37.7 (8.1)	
CES-DSF			0.02
0–4	57.4	41.2 (6.9)	
4 +	42.6	37.5 (8.3)	
Rating of oral health			0.01
Poor and fair	63.1	37.7 (8.2)	
Good and excellent	36.9	42.9 (5.6)	
*Oral hygiene behaviors*			
Brushing teeth frequency			0.88
Less than twice a day	25.4	38.9 (7.5)	
Twice a day or more	74.6	39.9 (7.9)	
Flossing frequency			0.54
Less than once a day	55.6	39.9 (7.6)	
Once a day or more	44.4	39.4 (8.0)	

Table 2 shows the Spearman correlations of cognitive and oral health status variables and GOHAI at T0. Two cognitive variables, fears and worries were significantly correlated with GOHAI: higher scores on fears (less fear) and higher scores on worries (less worries) were associated with higher GOHAI scores. Neither GI or PS were associated with GOHAI. Missing teeth and dry mouth were significantly associated with GOHAI. As much of the literature shows, more missing teeth and worse dry mouth were associated with poorer OHRQOL. The oral hygiene variables, GI and PS, were not correlated with GOHAI.

**Table 2:** Bivariate Spearman correlation coefficients between General Oral Health Assessment Index (GOHAI) and behavioral/psychosocial and oral health status variables at T0 (n = 331)

	Spearman Coefficient GOHAI T0	*p* value
Self-efficacy	0.10	0.07
Fears	0.24	< 0.001
Intentionality	0.05	0.35
Locus of control	0.01	0.85
Oral health norms	0.00	0.89
Worries	0.29	< 0.001
Sugar intake	− 0.07	0.18
Dry mouth	− 0.20	< 0.01
Missing Teeth	− 0.15	< 0.01
Gingival Index	− 0.07	0.23
Plaque Score	− 0.06	0.31

**Fig. 2:**
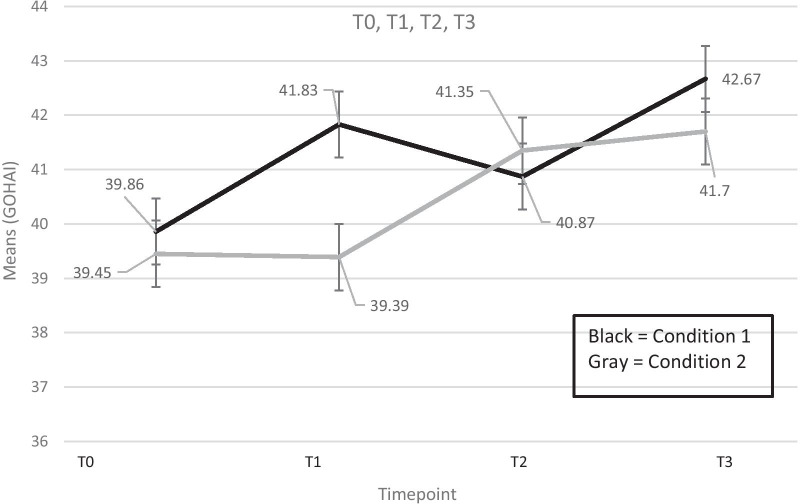
Mean (sd) scores for the General Oral Health Assessment Index, T0, T1, T2, T3. T0 = Baseline assessment; T1 = assessment at one month post intervention 1; T2 = assessment at one month post intervention 2; T3 = assessment at 6 months post T2 assessment

As stated above, mean GOHAI at T0 was 39.7 (sd = 7.8) and increased over time to 40.7 (sd = 8.2) at T1, to 41.1 (sd = 7.8) at T2 to 42.3 (sd = 7.1) at T3. Figure 2 presents the unadjusted mean GOHAI scores over time by intervention. For the Condition 1 sequence (one-on-one AMI intervention followed by the campaign), participants improved on GOHAI from T0 to T1 and appear to have significantly better scores at T1 than those in Condition 2. Those in Condition 1 stayed about the same after the campaign intervention at T2 and improved slightly at T3 and, again, appear to have significantly higher scores at T3 compared to those in Condition 2. Those in Condition 2 (campaign followed by the one-on-one AMI intervention) stayed the same from T0 to T1 but improved from T1 to T2 and improved slightly from T2 to T3.

Table 3 presents the trimmed model of the results of the GLMM analysis of the effects of time, intervention, sociodemographic, cognitive and oral health status variables. The effect of time was significant for the T0–T3 comparison, that is, GOHAI improved significantly over time from T0 to T3 (*p* = 0.01), but the differences in scores from T0-T1 and T0-T2 scores were not significant. The time by intervention interactions were not significant and were removed from the analysis. Hispanics had higher GOHAI scores compared to Blacks (*p* < 0.001) and those with more diagnoses that limit daily activities had significantly lower GOHAI scores. Oral health rating was significantly associated with GOHAI—those with worse oral health rating had worse GOHAI score (*p* < 0.001). Those with higher CESD-SF scores also had worse GOHAI (*p* = 0.04). Two cognitive/emotional variables, worries and self-efficacy, were significantly related to GOHAI. For Worries, those with better scores on the worries scale had better GOHAI scores compared to those with worse scores on worries (*p* < 0.001). For self-efficacy, those had a higher belief in their own ability to control their oral health had higher GOHAI scores (*p* = 0.01). Two oral health status variables, missing teeth and dry mouth were significantly related to GOHAI. More missing teeth (*p* < 0.001) and more dry mouth symptoms (*p* = 0.04) were significantly related to GOHAI. Neither GI nor PS was associated with GOHAI.

**Table 3:** General Linear Mixed Models multivariate analysis of the General Oral Health Assessment Index with demographic characteristics, cognitive/behavioral variables and oral health status variables

Effect	Estimate	Standard error	P level|	95% confidence interval, lower	95% confidence interval upper
Intercept	31.11	1.87	< 0.0001	27.43	34.79
Condition 1 (Condition 2 as reference)	0.57	0.56	0.31	− 0.52	1.66
Time 1 versus T0 as reference	0.20	0.42	0.64	− 0.62	1.02
Time 2 versus T0 as reference	0.17	0.46	0.71	− 0.74	1.08
Time 3 versus T0 as reference	1.25	0.48	0.01	0.31	2.18
Race (Hispanic vs Blacks as reference)	3.02	0.69	< 0.0001	1.66	4.38
Race (White & others vs Blacks as reference)	0.74	0.85	0.39	− 0.94	2.41
Diagnosis that interfere (1 + vs 0 as reference)	− 1.90	0.60	0.01	− 3.07	− 0.73
Oral health rating (Good and excellent vs poor and fair as reference)	2.98	0.58	< 0.0001	1.85	4.12
CESD (4 + vs less than 4 as reference)	− 1.21	0.58	0.04	− 2.35	− 0.08
Self-efficacy	0.93	0.38	0.01	0.19	1.68
Worries	2.48	0.30	< 0.0001	1.90	3.06
Dry mouth	− 0.70	0.12	< 0.0001	− 0.93	− 0.47
Missing teeth	− 0.10	0.03	0.01	− 0.17	− 0.032

## Discussion

This study assessed Oral Health Related Quality of Life (OHRQOL) among independent, community-dwelling older adults and disabled adults living in subsidized low-income senior housing. As is found in other community-based studies, the participants reported relatively good OHRQOL with a mean of 39.7 (sd = 7.8) of a possible 48. The distribution of scores was skewed to positive scores at each time point. Although many community-based studies report significant associations between OHRQOL and age, education, minority status and socioeconomic status, this study only found race/ethnicity to be important in the multivariate analysis. Hispanics had better GOHAI scores over time compared to Black participants. However, Whites did not have better OHRQOL compared to Blacks which other studies usually find. The sample consisted of a majority of Hispanic participants which may account for this result. Lack of associations between sociodemographic characteristics and GOHAI may be the result of having a somewhat homogeneous sample of vulnerable adults.

GOHAI improved significantly from the beginning of the study (T0) to T3, approximately 18 months after T0. Improvement occurred among all participants regardless of condition. The interventions did not appear to have a differential impact on GOHAI. Our previous papers [42, 43] have shown that both interventions resulted in significant improvements in GI and PS and that the AMI intervention was more effective in improving GI and PS over time. The campaign intervention did not seem to add much to improvement in GI or PS after the administration of the AMI.

Oral health, general health and mental health status were significantly related to GOHAI. As found in other studies, dry mouth symptoms and number of missing teeth were important determinants of GOHAI. More symptoms of dry mouth and more missing teeth were associated with worse GOHAI scores. Number of missing teeth was highly significant in the multivariate analysis. These clinical oral health status factors should be addressed in future interventions to improve OHRQOL through replacement of missing teeth and a review of medications that might cause dry mouth symptoms. Perceived oral health status was strongly and significantly related to GOHAI. This finding would be expected as perceived oral health is often used as a comparison measure to assess the validity of OHRQOL measures.

The general health status measure also was significantly related to GOHAI. Those with more physical limitations, measured by the number of diagnoses that interfere with daily activities, had worse GOHAI scores. It is likely that these individuals had more difficulty in caring for their teeth resulting in impacts on chewing, eating and appearance. Adaptive devices and strategies for these individuals could improve their OHRQOL.

Mental health status, measured by the CESD-SF, was significantly associated with GOHAI. This is not surprising since both indicators are measures of well-being. Those who had more depressive symptoms had more impacts on GOHAI. Depressive symptoms were common in this sample and should be addressed in oral health interventions aimed at improving OHQOL.

One cognitive variable was significantly related to OHRQOL. More worries about oral hygiene self-management was related to lower GOHAI. One item in the GOHAI specifically asked “How often were you worried or concerned about the problems with your teeth, gums or dentures?” and it is a high impact area with 42.3% of the sample replying “always, sometimes or never”. Therefore, we would expect that the worries scale would be associated with GOHAI. However, this finding demonstrates the importance of “worry” as an underlying construct in OHRQOL.

Overall, the findings show that participants in the study had good OHRQOL as measured by the GOHAI and both interventions improved GOHAI. While the interventions were associated with improvements in clinical oral hygiene outcomes, GI and PS, these clinical oral hygiene improvements were not related to GOHAI. GOHAI was a secondary outcome. Interventions aimed at improving oral hygiene might not be effective in improving OHRQOL unless explicitly addressing the OHRQOL dimensions that the GOHAI measures. Furthermore, a longer observation period might be necessary to detect changes in OHRQOL.

GOHAI may not be as sensitive to change as other measures of OHRQOL which could account for our findings that GOHAI did not change by type of intervention. However, an early study did show that GOHAI was sensitive to change in a health promotion intervention [44]. Several authors have used GOHAI as outcomes in clinical trials and found significant changes over time. For example, Jonsson and colleagues investigated the effects of periodontal surgery on OHRQOL [14]. The clinical sample had poorer overall GOHAI scores (mean score of 43.4 (sd = 8.8) of a possible 60) than our sample. The participants in the experimental group had a significant improvement of 1.8 (95% CI of 0.3 to 3.3) although differences in the GOHAI compared to the control group were not significant. As with the Jonsson study [14], the present study was not powered on the basis of GOHAI but rather on the primary outcomes of GI and PS. Future studies should consider OHRQOL measures when calculating sample size even when OHRQOL is a secondary outcome measure.

The results suggest that either intervention would be effective in future studies of quality of life outcomes in oral hygiene interventions. The AMI is more time-intensive and requires training of counsellors and the identification of areas of concern from the survey. The community-based intervention has the advantage of engaging community members in the intervention but these volunteers also required substantial training and the development of health fairs. The community intervention also has the advantage of potentially being able to reach larger numbers of people. Each of these interventions can be adapted or shaped to meet the needs of targeted groups. The trade-offs between the interventions need to weighed against the resources and goals of the study or program.

### Limitations of the study

The GOHAI was skewed to positive measures and the mean and median values of GOHAI among the participants was relatively high. There may have been a ceiling effect that limited the ability to detect meaningful changes in the GOHAI. The interventions did address worries, an area that was significantly associated with GOHAI, but other dimensions of the GOHAI, such as chewing, eating and social limitations, were not addressed. The interventions could be expanded to include more attention to the underlying dimensions of OHRQOL.

## Conclusions

The participants reported relatively good oral health related quality of life and improved significantly over time. Improvement occurred among all participants regardless of condition, suggesting that either intervention would be effective in future studies. This study adds to the expanding literature on oral health related quality of life and demonstrates the feasibility of using oral health related quality of life in oral health clinical trials.

## Data Availability

Data are available from the authors upon reasonable request.
